# Zika Virus with Increased CpG Dinucleotide Frequencies Shows Oncolytic Activity in Glioblastoma Stem Cells

**DOI:** 10.3390/v12050579

**Published:** 2020-05-25

**Authors:** Ivan Trus, Nathalie Berube, Peng Jiang, Janusz Rak, Volker Gerdts, Uladzimir Karniychuk

**Affiliations:** 1 Vaccine and Infectious Disease Organization-International Vaccine Centre (VIDO-InterVac), University of Saskatchewan, Saskatoon, SK S7N 5E3, Canada; ivan.trus@usask.ca (I.T.); nab602@mail.usask.ca (N.B.); volker.gerdts@usask.ca (V.G.); 2 Department of Cell Biology and Neuroscience, Rutgers University, Piscataway, NJ 08854-8082, USA; peng.jiang@rutgers.edu; 3 The Research Institute of the McGill University Health Centre, Montreal, QC H3H 2R9, Canada; janusz.rak@mcgill.ca; 4 Department of Veterinary Microbiology, Western College of Veterinary Medicine, University of Saskatchewan, Saskatoon, SK S7N 5B4, Canada; 5 School of Public Health, University of Saskatchewan, Saskatoon, SK S7N 2Z4, Canada

**Keywords:** CpG recoding, oncolytic virus, Zika virus, glioblastoma, stem cells, CAM, egg

## Abstract

We studied whether cytosine phosphate–guanine (CpG) recoding in a viral genome may provide oncolytic candidates with reduced infection kinetics in nonmalignant brain cells, but with high virulence in glioblastoma stem cells (GSCs). As a model, we used well-characterized CpG-recoded Zika virus vaccine candidates that previously showed genetic stability and safety in animal models. In vitro, one of the CpG-recoded Zika virus variants had reduced infection kinetics in nonmalignant brain cells but high infectivity and oncolytic activity in GSCs as represented by reduced cell proliferation. The recoded virus also efficiently replicated in GSC-derived tumors in ovo with a significant reduction of tumor growth. We also showed that some GSCs may be resistant to Zika virus oncolytic activity, emphasizing the need for personalized oncolytic therapy or a strategy to overcome resistance in GSCs. Collectively, we demonstrated the potential of the CpG recoding approach for oncolytic virus development that encourages further research towards a better understanding of host–tumor–CpG-recoded virus interactions.

## 1. Introduction

Cytosine–phosphate–guanine (CpG) dinucleotide frequencies are suppressed in vertebrate genomes and most RNA viruses [1,2]. The rational increase of CpG dinucleotide numbers in viral genomes has the potential to become a cutting-edge approach for vaccine development and an alternative to traditional live attenuated vaccines. The concept is to increase the number of CpG dinucleotides in an RNA viral genome while retaining the amino acid composition of encoded proteins that leads to impaired infection but robust protective host immune responses. Mechanistically, it has been demonstrated that cellular Zinc-finger antiviral protein (ZAP) targets recoded viruses by specifically binding to genomic regions enriched in CpG dinucleotides [3,4]. Subsequently, synergy or complementation of ZAP function by oligoadenylate synthetase 3, RNase L, and cytoplasmic protein KHNYN inhibits replication of viruses containing an elevated number of CpG dinucleotides [5,6]. Efficacy of the CpG-recoded influenza virus vaccine has been demonstrated in mice; we also showed full protection evoked by CpG-recoded Zika virus (ZIKV) vaccine candidates in mice challenged with lethal heterologous ZIKV [7].

Zika virus emerged in the Americas in 2015, evoking great concern around the world with fetal death, microencephaly, severe brain lesions, and developmental abnormalities in fetuses and offspring. Paradoxically, neurotropic ZIKV was explored as an oncolytic virus. The idea was to apply ZIKV in a surgical area of the brain after glioblastoma removal to suppress the growth of glioblastoma stem cells (GSCs) and tumor recurrence. The authors showed oncolytic activity of ZIKV in GSCs in vitro and in a glioblastoma mouse model [8,9]. The approach is relevant because patients suffer inevitable relapses after glioblastoma surgery, and accumulating evidence indicates that GSCs play a central role in tumor recurrence [10,11]. Glioblastomas rarely metastasize beyond the brain, and patients usually suffer a recurrence within proximity of the surgical zone [12] that supports the proposed locally-targeted therapeutic approach. Also, glioblastoma therapies with local delivery of viral vectors showed feasibility [13]. Later, attenuated ZIKV vaccine candidate—with 10 nucleotide deletions in 3′ UTR—was re-purposed for oncolytic therapy showing efficacy in GSCs in vitro and in the mouse model [14]. Moreover, ZIKV oncolytic activity was demonstrated in embryonal central nervous system tumor xenografts and in dogs with large brain tumors [15,16].

These pioneering ZIKV studies, previous findings that ZAP—the host protein targeting CpG-enriched regions in recoded viruses—is underrepresented in some cancer cells [17], and advances in the field of oncolytic virotherapy [18,19,20,21,22] inspired us to test whether CpG recoding in viral genomes has an oncolytic potential. Our main goal was to study whether CpG-recoding in a viral genome may provide an oncolytic candidate with reduced infection kinetics in healthy brain cells but with high virulence in GSCs. As a model, we used ZIKV: first, we tested the oncolytic activity of CpG-recoded ZIKV variants in two human primary GSCs in vitro; second, we established an in ovo glioblastoma model and tested how CpG-recoded ZIKV variants affect the growth of GSC-derived tumors.

## 2. Materials and Methods

### 2.1. Cell Cultures

C6/36 cells (ATCC #CRL-1660, Rockville, MD, USA) were maintained in minimum essential medium (MEM; Sigma #M4655, St. Louis, MO, USA) supplemented with 10% fetal bovine serum (FBS; Sigma #12103c) and 1x P/S (Penicillin-Streptomycin; Gibco #15140-122, Grand Island, NY, USA). VERO E6 cells (ATCC #CRL-1586, Rockville, MD, USA) were maintained in Dulbecco’s modified Eagle’s medium (DMEM) supplemented with 3% FBS, 1x P/S, and 2.67 mM Sodium Bicarbonate (Gibco #25080-094, Grand Island, NY, USA). The human microglial HMC3 cells (ATCC #CRL-3304, Rockville, MD, USA) were maintained in MEM supplemented with 10% FBS and 1x P/S. The human NPCs were differentiated from human induced pluripotent stem cells that were reprogrammed from fibroblasts obtained from a healthy individual [23,24] (the University of Saskatchewan’s Biomedical Research Ethics Board Number, #17-181, 9 July 2019); NPCs were cultured in medium consisting of 50% DMEM/F12 (HyClone #SH3002301, Logan, UT, USA) and 50% neurobasal medium (Gibco #21103-049, Grand Island, NY, USA) containing 1x B27-RA, 1x N-2 (Gibco #17502-048, Grand Island, NY, USA), 1x P/S, 20 ng/mL basic fibroblast growth factor (bFGF, PeproTech #100-18B, Rocky Hill, NJ, USA), 2 µM SB431542 (Reprocell Stemgent #04-0010-10, Cambridge, MA, USA), 10 ng/mL leukemia inhibitory factor (PeproTech #300-05, Rocky Hill, NJ, USA), 3 µM CHIR99021 (StemCell Technologies #72052, Vancouver, BC, Canada), and 10 µM Y-27632 (Tocris Bioscience #12-541, Bristol, UK) [25]. For NPCs, plates were precoated with growth factor-reduced Matrigel (BD Biosciences #354230, San Jose, CA, USA). Previously well-characterized GSC 157 and 528 (obtained from patients with high-grade gliomas and characterized as a proneural and mixed subtype, respectively) [26,27] were cultured in DMEM/F12 with 1x B27-RA (Fisher Scientific #12-587-010, Ottawa, ON, Canada), 1x P/S, 3 IU/mL Sodium Heparin (Fisher Scientific #H19, Ottawa, ON, Canada), 20 ng/mL bFGF, and 20 ng/mL epidermal growth factor (StemCell Technologies #78006, Vancouver, BC, Canada). Low passages (<10) of NPC and GSC cells were used in the study. Cells were cultured at +37 °C (C6/36 cells were cultured at +28 °C) in a 5% CO_2_ humidified incubator. For detachment of C6/36 cells, we used cell scrappers (Fisher Scientific #08-100-242, Ottawa, ON, Canada), HMC3 and VERO—trypsin-EDTA (Gibco #25200-072, Grand Island, NY, USA), NPCs—TrypLE (Gibco #LS12604021, Grand Island, NY, USA), and GSCs—Accumax (StemCell Technologies #07921, Vancouver, BC, Canada).

### 2.2. Design and Recovery of CpG-Recoded ZIKV Variants

**Figure 1:**
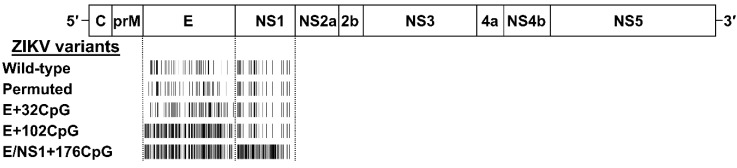
The genome of Zika virus (ZIKV) and the CpG (cytosine–phosphate–guanine)-recoding strategy. ZIKV genomic regions encoding envelope (E) and non-structural 1 (NS1) proteins were recoded to increase the number of CpG dinucleotides. A barcode schematically represents the number of CpG dinucleotides. The actual number of CpG dinucleotides are in Supplementary Table S1.

In silico recoding and recovery of CpG-modified ZIKV variants were previously described [7]. The MUTATE SEQUENCES program in the SSE 1.3 software package (Peter Simmonds, University of Oxford, Oxford, UK) [28] was used to modify the sequence of the contemporary Asian ZIKV H/PF/2013 strain [GenBank: KJ776791.2] [29] and to generate variants with increased CpG numbers in regions encoding envelope (E) and nonstructural 1 (NS1) proteins (Figure 1). Introduced nucleotide mutations did not alter the translated viral proteins. We also renormalized frequencies of uracil-phosphate-adenine (UpA) dinucleotides in recoded ZIKV variants to the initial level. Recoded variants showed a modest reduction in codon pair bias scores in the E and NS1 genomic regions or minimal changes in the complete ORF [7].

To ensure sequence disruption did not damage or destroy the unknown replication element(s), we designed a permuted control (Figure 1); the sequence region was permuted using the CDLR method in the SSE software package [30,31,32].

To recover ZIKV variants we used infectious subgenomic amplicons (ISA) [29,33,34] as previously described [7]. Recoded fragments were de novo synthesized (GenScript, Piscataway, NJ, USA), amplified with high fidelity PCR (Invitrogen Platinum PCR SuperMix, High Fidelity #12532016, Carlsbad, CA, USA), transfected into C6/36 *Aedes albopictus* mosquito cells at +37 °C for 12 h, and incubated for seven days at +28 °C [33]. Media from virus-negative C6/36 cells were used as a control for transfection. After passaging twice in C6/36 cells, cell culture media containing ZIKV were centrifuged (12,000× *g*, 20 min, +4 °C), and frozen (−80 °C). Viral titers were quantified in triplicate in VERO cells with the endpoint dilution assay described below.

All recovered ZIKV variants showed stability of de novo introduced CpG dinucleotides after ten passages in VERO cells and infection in neonatal mice [7]. All virus stocks and cell cultures were free of mycoplasma contaminations as confirmed by a PCR Detection Kit (Sigma #MP0035, St. Louis, MO, USA).

### 2.3. Replication Phenotypes of CpG-Recoded ZIKV Variants in Vitro

We evaluated the viral replication kinetics in cell cultures of human origin (HMC3, NPCs, GSC 157, and GSC 528) as previously described [7]. Cells in suspension were inoculated at multiplicity of infection (MOI) of 0.01 in 100 µL of appropriate cell culture medium. Eppendorf tubes with inoculated cells were incubated at +37 °C for 1 h and shaken gently every 10 min. Then, cells were washed three times with serum-free media and seeded in 96-well plates. Wells were first coated with growth factor-reduced Matrigel and prefilled with 150 µL of cell culture medium. Then, 50 µL of cell suspension was added on top to obtain a resulting concentration of 4 × 10^4^ (HMC3) or 10^5^ (NPC, GSC 157, and GSC 528) cells per well. Plates corresponding to different experimental time points were infected at the same initial time. Mock-infected cells were included as controls in each plate.

Infected plates were incubated (5% CO_2_, +37 °C) until the sampling time point. Then, supernatants were collected, clarified (2000× *g*, 5 min, +4 °C), and frozen (−80 °C) until subsequent infectious virus quantification with the endpoint dilution assay described below [7,35,36,37,38,39]. Cell culture media were serially diluted fivefold in four replicates starting from 1:5, and 50 µL of each dilution was added to confluent VERO cell monolayers cultured in 96-well plates. Dilutions were made in complete cell culture media. After 2 h, 150 µL of fresh media was added to each well. The cells were incubated for seven days. After washing and drying, the plates were kept at −20 °C at least for 2 h or until use. Cell fixation and staining with virus-specific 4G2 Abs were done, as previously described [7,35,36,37,38,39]. Fifty percent tissue culture infective dose (TCID_50_) endpoint titers were calculated by the Spearman–Kärber formula and expressed in a decimal logarithm. Media from mock-inoculated cells were used as negative controls.

After the supernatant collection, the plate with infected cells was dried and frozen (−20 °C). Plates were stained with anti-pan flavivirus E protein monoclonal 4G2 antibodies (Abs; ATCC #HB-112, Rockville, MD, USA), and infected cells were counted in the well with bright-field microscopy at 200× magnification and expressed per cm^2^ [7,35]. Cell culture supernatants and fixed plates were collected at 0–5 days post-inoculation, with three technical replicates and three biological replicates per time point for each ZIKV variant.

### 2.4. Cell Proliferation Assay

Cells in suspension were inoculated at an MOI of 1 in 100 µL of appropriate cell culture medium. Eppendorfs with inoculated cells were incubated at +37 °C for 1 h and shaken gently every 10 min. Then, cells were washed with media and seeded in 96-well plates. Wells were first prefilled with 50 µL of cell culture medium, and 50 µL of cell suspension was added on top to obtain a resulting concentration of 5 × 10^3^ cells per well. Plates corresponding to different experimental time points were infected at the same initial time. Mock-infected cells were included as controls in each plate.

On days 0, 1, 3, 5, and 7, cell proliferation was analyzed with the CellTiter-Glo Luminescent Assay (Promega #G7571, Madison, WA, USA) according to the manufacturer’s instructions. For analysis, 96-well black plates (PerkinElmer #6005660, Waltham, MA, USA) and CellTiter-Glo reagent were equilibrated to room temperature (+22 °C, 30 min). Then, CellTiter-Glo reagent was added to each well, and plates were placed on an orbital shaker (+22 °C, 12 min). Luminescence was quantified on a microplate reader (Promega GloMax Explorer, Madison, WA, USA). All data were normalized to day 0 and expressed as relative cell proliferation.

### 2.5. Chicken Chorioallantoic Membrane (CAM) Assay for GSCs

Chicken CAM assays are commonly used in cancer research [40,41]. Here, to develop the in ovo model for glioblastoma, we implanted GSCs on chicken CAM.

Experiments were performed following the Canadian Council on Animal Care guidelines for humane animal use and were approved by the University of Saskatchewan’s Animal Research Ethics Board (#004CatA2017, 4 June 2019). Fertilized eggs of Lohmann Selected Leghorn layers (LSL-Lite, Lohmann, Cuxhaven, Germany) were placed in an incubator (GQF #1502, Savannah, GA, USA) and maintained at 37.8 ± 0.1 °C and 50 ± 2% of relative humidity with turning every 2 h. The day on which eggs were placed in the incubator was considered as day 0 of embryonic development (ED).

On ED 6, eggs were candled and infertile eggs were excluded. Viable eggs (94.1%) were turned into a horizontal position and the upper surface was marked with a pencil. The automatic rotation was turned off starting from this day. On ED 7, windowing and CAM dropping were done as previously described with a rotary tool, generating an artificial air sac [42,43,44,45]. The created window was closed with a semipermeable adhesive film (3M Tegaderm Roll #16004, St. Paul, MN, USA). Eggs were placed back in the incubator, and relative humidity was increased to 54 ± 2%.

On ED 10, we verified egg viability and implanted GSC as previously described [46,47]. First, the adhesive film was removed with sterile scissors. Then, the CAM blood vessel was damaged by squeezing with forceps until mild bleeding was visually observed, and a sterile Teflon O-ring (the O-Ring Store #AS568-010 TEF010, Clarkson, WA, USA) was placed with sterile forceps on CAM with the ruptured vessel in the middle [48]. One million live cells were resuspended in 50 µL of fresh ice-cold cell culture medium containing 25% of Matrixgel HC (Corning #354262, Corning, NY, USA). Then, an ice-cold cell suspension was slowly placed inside the O-ring (25 µL/min) to allow gel solidification and prevention of leakage of the cell suspension outside the ring.

The window was closed again with the adhesive film, and eggs were placed in the egg incubator (37 °C) with windows facing the top.

On ED 19, viable embryos were placed at +4 °C for 2 h. Macro photos of opened eggs with tumors were taken with a stereomicroscope (Leica #M80 with #MC170 HD digital camera, Wetzlar, Germany), and tumor volumes were calculated according to Hagedorn et al. [49]:

(1) $$V=\frac{4}{3}\pi {\left(\frac{1}{2}\sqrt{(d_{1}d_{2}}\right)}^{3},$$

where *d*_1_ and *d*_2_ are the diameters of the tumor measured with ImageJ 1.51r (NIH, Bethesda, MD, USA).

Tumors were separated from surrounding tissues with sterile forceps and scissors and placed in 10% paraformaldehyde for hematoxylin and eosin (H&E) staining, embedded in NEG-50 medium (Thermo Fisher Scientific #6502, Waltham, MA, USA), and snap-frozen (−80 °C) for immunohistochemistry or preserved in PCR-grade Eppendorfs (−80 °C) for ZIKV quantification.

### 2.6. Oncolytic Phenotypes of CpG-Recoded ZIKV Variants in the Glioblastoma CAM Model

We evaluated ZIKV loads and tumor growth reduction in a CAM assay. For inoculation, cells were resuspended in 200 µL of appropriate cell culture media as described above; media contained ZIKV variants at MOI of 0.25. Eppendorf tubes with cells were incubated at +37 °C for 1 h and shaken gently every 10 min. Afterward, cells were washed three times with media and seeded on CAM (10^6^ cells/egg) at ED 10 as described above. Eggs were placed in incubators until the sampling time point at ED 19.

At sampling, the size of tumors was measured as described above. To assess size reduction in ZIKV-infected tumors, the average size of the tumors in the mock-infected group was divided by the average size of ZIKV-infected tumors.

### 2.7. RNA Extraction and Reverse Transcriptase Quantitative Polymerase Chain Reaction Assay (RT-qPCR)

Single-use scalpel blade and sterile forceps were used to separate 9–54 mg of tumor tissue. Tissue samples were weighed on analytical balances, and after adding 0.6 mL of lysis buffer, were homogenized using RNase-free stainless steel beads and TissueLyser II (QIAGEN, Hilden, Germany) operating for 5 min at 25 Hz. Then, RNA extraction was continued with the PureLink RNA Mini Kit (Invitrogen #12183025, Carlsbad, CA, USA) according to the manufacturer’s instructions.

ZIKV-specific SYBR Green-based one-step RT-qPCR was used for ZIKV RNA quantification in tumors [50]. PCR reactions were conducted on the StepOne Plus platform (Applied Biosystems, Foster City, CA, USA) and analyzed using StepOne 2.3 software (Applied Biosystems, Foster City, CA, USA). The reaction mixture (20 µL) consisted of 10 µL 2× SensiFAST SYBR Hi-ROX One-Step Mix (Bioline #BIO-73005, London, UK), 0.4 µL RiboSafe RNase Inhibitor, 0.2 µL reverse transcriptase, 0.8 µL (400 nM) of each primer (ZIKV-F10287: 5′-AGGATCATAGGTGATGAAGAAAAGT-3′; ZIKV-R10402: 5′-CCTGACAACACTAAGATTGGTGC-3′), 3.8 µL nuclease-free water, and 4 µL RNA template. A reverse transcription step of 10 min at 45 °C and an enzyme activation step of 2 min at 95 °C were followed by 40 amplification cycles (5 s at 95 °C and 34 s at 60 °C). RNA (10238–10444 = 207 nt amplicon) from a stock of the ZIKV PRVABC59 strain [GenBank: KU501215.1] was used to generate a standard curve and quantify viral RNA loads. The standard curve had a wide dynamic range (10^2^–10^9^ copies/reaction) with a high linear correlation (*R^2^* = 0.9997) between the cycle threshold (*Cq*) value and template concentration. The slope of the standard curve (−3.4351) corresponded to the 95.5% reaction efficiency level. PCR values were corrected for tissue weights and upon logarithmical transformation expressed as ZIKV RNA genome copies per gram. In all PCR tests, we used VERO cell culture media containing ZIKV as a positive PCR control. As a negative control, we used samples from mock-inoculated cells. Strict precautions were taken to prevent PCR contamination. Aerosol-resistant filter pipette tips and disposable gloves were used. Kit reagent controls were included in every RNA isolation and PCR run.

### 2.8. Histopathology and Immunohistochemistry (IHC)

Tumors collected from chicken eggs were fixed in formalin for subsequent H&E staining.

For immunohistochemistry, staining was performed as previously described [7,35,51] with some modifications. Briefly, slide chambers were covered with Matrigel and seeded with cells. Slide chambers after reaching cell confluence or 10 µm cryosections of tumors were fixed in 10% buffered formalin at +4 °C for 15 min. After treatment with 1% H_2_O_2_ and 1% Triton X-100 (20 min, RT), chambers and tissue sections were incubated with primary monoclonal Abs (mouse anti-ZIKV: ATCC #HB-112, 1/20, Rockville, MD, USA; mouse anti-TGM2: Thermo Fisher Scientific #MA5-12739, 1/50, Waltham, MA, USA; rat anti-SOX2: eBioscience #14-9811-80, 1/50, San Diego, CA, USA) for 1 h at +37 °C. Then, chambers and cryosections were incubated with horseradish peroxidase-conjugated reagents (ZIKV and TGM2: anti-mouse Envision HRP labelled polymer, Agilent #K4001, Palo Alto, CA, USA; SOX2: rabbit anti-rat, Abcam #ab6734, 1/200, Cambridge, UK) following the Lab Vision Ready-To-Use AEC Substrate System (Abcam #ab64252, Cambridge, UK) according to the manufacturer’s instructions. Subsequently, tissues were counterstained with hematoxylin. Examination and imaging were performed under a microscope (Leica #DM2000 LED with #MC170 HD digital camera, Wetzlar, Germany).

### 2.9. Statistical Analysis

Zika virus infectious titers in cell culture supernatants from NPC, HMC3, and GSCs and cell proliferation data were assessed with non-parametric analysis of variance after aligned rank transformation [52]. Results of statistical analyses are provided in text (main effects), figures or figure legends (interaction effects in a two-way model).

Zika virus loads and tumor growth in ovo were compared using the Kruskal–Wallis H test and Dunn’s multiplicity-adjusted post-test.

Results were considered significantly different when *p* < 0.05.

## 3. Results

### 3.1. CpG-Recoded ZIKV Variants Show Reduced Infection Kinetics in Nonmalignant Human Brain Cells and Distinct Oncolytic Activity in Different Glioblastoma Stem Cells in Vitro

**Figure 2:**
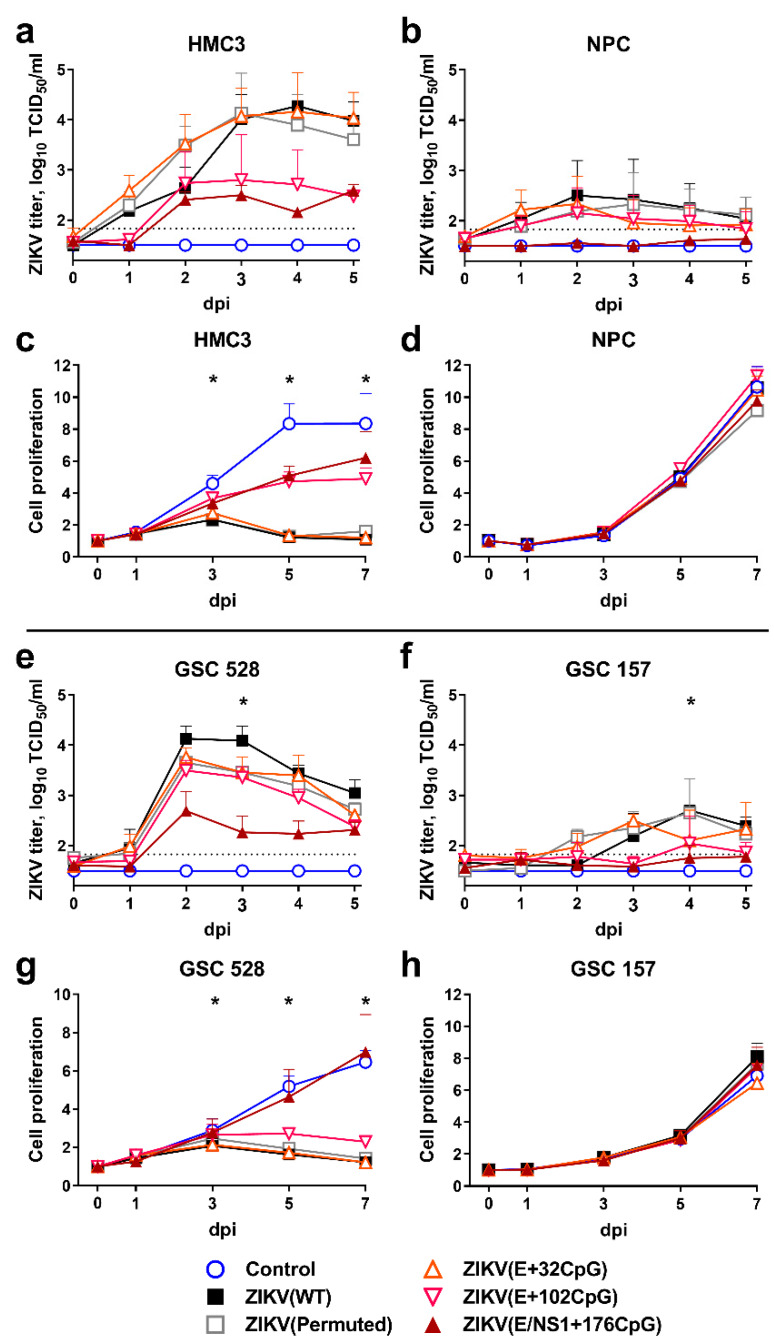
Infection kinetics in nonmalignant human brain cells (HMC3 (**a**) and NPC (**b**)) and tumor glioblastoma stem cells (GSC 528 (**e**) and GSC 157 (**f**)) after inoculation at multiplicity of infection (MOI) of 0.01. Cell culture supernatants in 96-well plates were collected and viral titers were measured using the endpoint dilution assay. The dotted line represents the limit of detection. Cell proliferation assay after inoculation of cells (HMC3 (**c**) and NPC (**d**), GSC 528 (**g**), and GSC 157 (**h**)) with MOI of 1. Whiskers represent the standard error of the mean (SE) from three biologically independent replicates with three technical replicates. “dpi”—days post-inoculation. The asterisk (*) indicates *p* < 0.05 vs. WT (**a**,**b**,**e**,**f**) and control (**c**,**d**,**g**,**h**): (**c**) WT and E+32CpG at 3–7 dpi, permuted control at 5–7 dpi; (**e**) E/NS1+176CpG at 3 dpi; (**f**) E+32CpG and E/NS1+176CpG at 4 dpi; (**g**) WT, permuted control, E+102CpG at 3–7 dpi.

We compared infection kinetics caused by WT and CpG-recoded ZIKV variants in HMC3 and NPCs representing human nonmalignant brain cells and in GSC 528 and GSC 157 representing human glioblastoma stem cells (Figure 2) [26,27].

Wild-type, permuted control, and the E+32CpG variant—the variant with the lowest CpG content among all recoded variants—showed similarly high infectious viral loads (*p* = 0.87–0.99) and kinetics in the HMC3 cell line (Figure 2a). In contrast, other CpG-recoded variants with the higher CpG content—ZIKV E+102CpG (*p* = 0.059) and ZIKV E/NS1+176CpG (*p* = 0.001; only 0.7 log_10_ above the detection limit)—showed reduced infectious titers (Figure 2a). All ZIKV variants, except ZIKV E/NS1+176CpG (*p* = 0.018), replicated more slowly in NPCs, producing low infectious titers (*p* = 0.96–0.99) (Figure 2b). The ZIKV NS1/E+176CpG variant—one with the highest CpG content among all recoded viruses—did not show infectious titers in NPCs (Figure 2b). Quantification of virus-positive cells was in accordance with the endpoint dilution assay (Supplementary Figure S1a,b).

Results of the proliferation assay of nonmalignant brain cells were in strong agreement with infection kinetics: HMC3 cells infected with both ZIKV E+102CpG and ZIKV E/NS1+176CpG showed high proliferation—close to the mock-infected control (*p* = 0.29–0.46; Figure 2c). In contrast, HMC3 cells infected with WT, permuted control, and ZIKV E+32CpG did not show proliferation (*p* < 0.001). Infection with any ZIKV variant did not affect the proliferation of NPCs (*p* > 0.99; Figure 2d).

Zika virus variants showed distinct infection phenotypes in different GSCs. In GSC 528, only the E/NS1+176CpG variant—the variant with the highest CpG content—showed a considerable reduction in infectious titers (*p* ≤ 0.002; Figure 2e) and in the number of ZIKV-infected cells (Supplementary Figure S1b). All other variants, including ZIKV E+102CpG—the variant with the second-highest CpG content, showed similar infection kinetics with high infectious titers (*p* = 0.15–0.44). In GSC 157, however, infection with all ZIKV variants resulted in infectious titers close to or below the detection limit (Figure 2f).

In agreement with infection phenotypes, all ZIKV variants (except ZIKV NS1/E+176CpG) considerably reduced proliferation of GSC 528 (*p* ≤ 0.005; Figure 2g). More resistant to infection, GSC 157 did not show changes in proliferation kinetics (*p* ≥ 0.19; Figure 2h).

In summary, while increasing the ZIKV genomic CpG content reduced infection kinetics in nonmalignant brain cells (Figure 2a,b), the recoded ZIKV E+102CpG variant showed oncolytic activity in glioblastoma stem cells as represented by high viral loads and reduced GSC proliferation. The in vitro oncolytic activity, however, was induced only in GSC 528 (Figure 2e).

### 3.2. Implantation of Human GSCs on CAM Leads to Tumor Growth

To further assess whether CpG-recoded ZIKV variants show oncolytic activity in GSC-derived tumors, we developed the CAM model.

**Figure 3:**
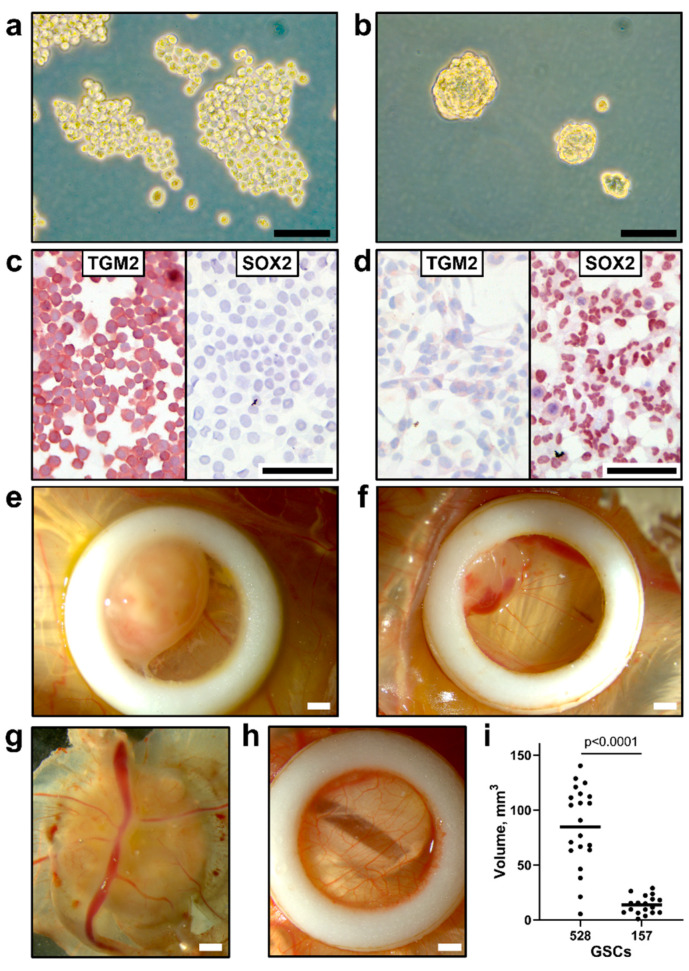
Morphology of glioblastoma stem cells (**a**: GSC 528; **b**: GSC 157) in vitro; phase-contrast microscopy. (**c**) Positive TGM2 and negative SOX2 staining in GSC 528. (**d**) Negative TGM2 and positive SOX2 staining in GSC 157. Morphology of GSC tumors (**e:** GSC 528; **f**: GSC 157) in in ovo cultures by bright-field microscopy. (**g**) Vascularization of a GSC tumor. (**h**) Intact chorioallantoic membrane in a control egg. (**i**) The volume of tumors formed at sampling (day 19 of embryonic development (ED); Mann–Whitney test). Scale bars are 0.1 (**a**–**d**) and 1 mm (**e**–**h**). Implantation efficiency of GSC cell cultures (GSC 528: *n* = 27, GSC 157: *n* = 26) on chicken embryo CAM (GSC 528: 96%, GSC 157: 88%) and egg viability at ED 19 (GSC 528: 92%, GSC 157: 93%) were comparable in both GSC models.

GSC 528 and GSC 157 had a different growth pattern and cell marker expression. In vitro, GSC 528 formed loose spheres (Figure 3a), while GSC 157 formed compact spheres that remained integrated after gentle pipetting (Figure 3b). We stained both cell types with SOX2 and TGM2 markers; these markers have been previously used to characterize GSCs 528 and 157 [27]. GSC 528 was positive for TGM2 (Figure 3c), but negative for SOX2. While SOX2 expression was previously described in GSC 528, the loss of this marker during passaging was also reported, which highlights a mixed composition of these cells [53,54]. In accordance with a previous report [27], GSC 157 showed no TGM2 and strong SOX2 expression (Figure 3d).

**Figure 4:**
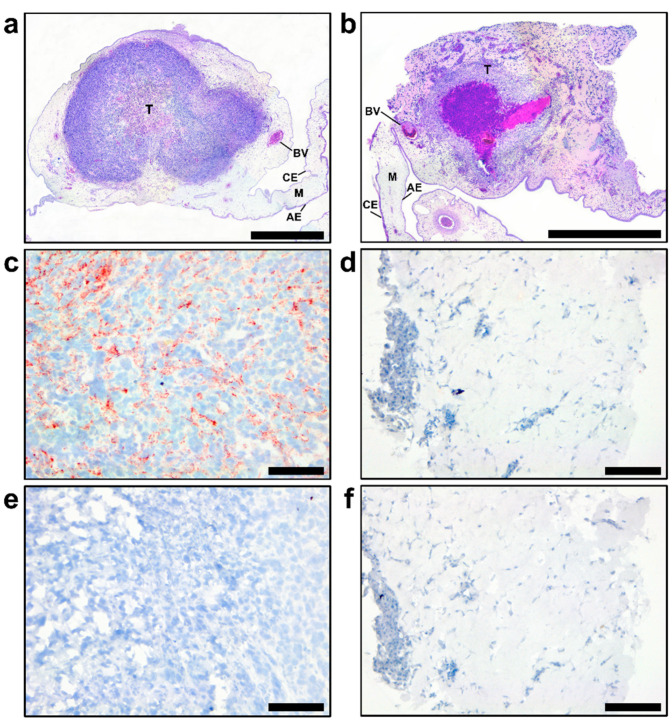
Hematoxylin and eosine staining in glioblastoma stem cells (GSC 528 (**a**) and GSC 157 (**b**) tumors at day 19 of embryonic development. CE: chorionic epithelium, AE: allantoic epithelium, M: intermediate vascularized mesenchyme, BV: blood vessel, and T: tumor. TGM2 (**c**: GSC 528; **d**: GSC 157) and SOX2 (**e**: GSC 528; **f**: GSC 157) protein expression in tumor cells; TGM2-positive staining is in red (**c**). Scale bars are 1 (**a**,**b**) and 0.1 mm (**c**–**f**).

In agreement with different in vitro growth patterns and distinct cell phenotypes, GSC 528 and GSC 157 showed different tumor formation phenotypes in ovo. Both cell types formed compact round-shaped solid tumors (Figure 3e,f) with the vascular network (Figure 3g); however, GSC 528 tumors were on average 6.2 times larger than GSC 157 tumors (Figure 3i). Histologically, GSC-derived tumors were vascularized (Figure 4a,b). GSC 528 tumors were encapsulated in the CAM mesenchyme, while GSC 157 had multiple nuclear-free zones with an unstructured background stained with eosin (Figure 4a,b). In accordance with in vitro staining, IHC in GSC 528 tumors showed expression of TGM2 (Figure 4c), while GSC 157 tumors lost the SOX2 marker (Figure 4d,f).

In summary, we demonstrated GSC growth and tumor formation in CAM. In accordance with distinct in vitro growth and protein marker expression, GSC 528 and GSC 157 showed different patterns of tumor growth and formation in ovo.

### 3.3. CpG-Recoded ZIKV Variants Show Distinct Oncolytic Activity in Different Glioblastoma Stem Cells in Ovo

After the in ovo model was established, we compared the infection kinetics and oncolytic activity of ZIKV variants in GSC-derived tumors. As in the previous mouse study [14], we used in ovo transplantation of GSCs pre-treated with ZIKV; this approach partially reproduces the prevention of glioblastoma recurrence in the clinical setting, where tumor is surgically removed and then chemotherapy and radiation are applied to eliminate residual malignant cells [55]. Because in vitro infection phenotypes caused by permuted control and ZIKV E+32CpG variants did not differ from the WT variant, we only focused on WT, ZIKV E+102CpG, and E/NS1+176CpG variants.

**Figure 5:**
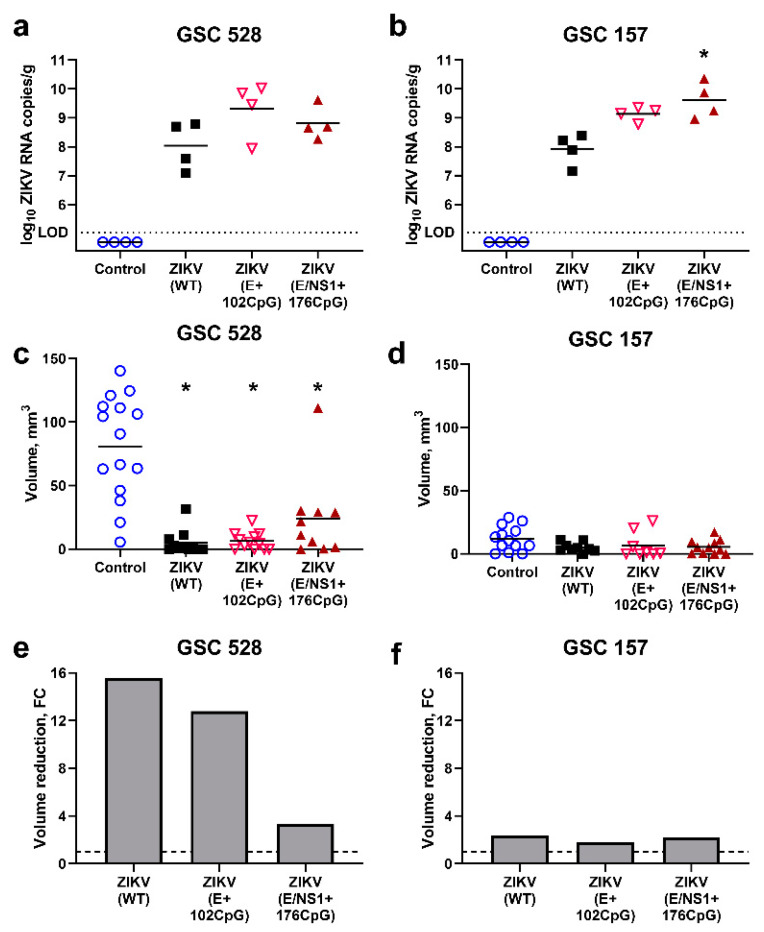
Zika virus (ZIKV) quantification in tumors ((**a**) glioblastoma stem cells (GSC) 528; (**b**) GSC 157). The dotted line (**a**,**b**) represents limit of detection. The volume of tumors inoculated with ZIKV variants (**c**: GSC 528; **d**: GSC 157). Relative reduction of tumors ((**e**) GSC 528; (**f**) GSC 157); FC: fold change. *: *p* < 0.05; tumor volumes in ZIKV groups were compared to volumes in the control group. The dashed line (**e**,**f**) represents the base tumor volume in the control group. Sampling was performed at day 19 of embryonic development.

Interestingly, both CpG-recoded variants showed higher (0.8–1.3 log_10_) viral loads than the WT variant in GSC 528 and 157 tumor tissues (Figure 5a,b).

**Figure 6:**
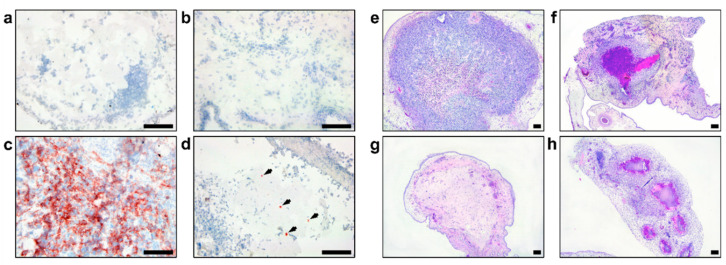
Immunohistochemistry of the Zika virus (ZIKV) antigen: (**a**) Isotype control staining of the glioblastoma stem cells (GSC) 528 tumor; (**b**) Mock-inoculated GSC 157 tumor; (**c**) GSC 528 inoculated with ZIKV E+102CpG; (**d**) GSC 157 inoculated with ZIKV E+102CpG (arrows). H&E staining of mock (**e**: GSC 528; **f**: GSC 157) and ZIKV-inoculated tumors (**g**: GSC 528 inoculated with ZIKV E+102CpG; **h**: GSC 157 inoculated with ZIKV E+102CpG). Scale bars are 0.1 mm.

The GSC 528 tumor size was considerably reduced—16 and 13 times—in ZIKV-WT (*p* < 0.0001) and ZIKV E+102CpG groups (*p* < 0.0001), respectively (Figure 5c,e). In the ZIKV E/NS1+176 CpG group, the tumor size reduction was lower—3.4 times—but still significant (*p* = 0.03; Figure 5c,e). Despite high viral titers in GSC 157 tumors (Figure 5b), the tumor size reduction was only 1.8–2.3 times in all groups (*p* = 0.22–0.50; Figure 5d,f). Zika virus antigens, phenotypical alterations, and size reduction in tumors were also revealed by IHC and H&E staining (Figure 6).

To summarize, all ZIKV variants replicated in GSC-derived tumors showed high viral titers. All ZIKV variants significantly reduced the growth of GSC 528-derived tumors; in contrast, GSC 157-derived tumors were resistant to oncolytic activity.

## 4. Discussion

Our main goal was to probe CpG recoding in a viral genome for the development of oncolytic candidates. We studied whether ZIKV variants with an increased CpG content have reduced infection kinetics in nonmalignant brain cells while retaining virulence in GSCs. In vitro, ZIKV E+102CpG and E/NS1+176CpG variants with the increased CpG content showed reduced infection kinetics in nonmalignant microglia cells; the proliferation activity of nonmalignant cells was also mostly not affected. In contrast, similar to the wild-type virus, the recoded ZIKV E+102CpG—the variant with the second-highest CpG content—showed oncolytic activity in GSCs 528 as represented by high viral loads and reduced cell proliferation. Next, we established, to our knowledge, the first CAM model for GSC-derived tumors and demonstrated high oncolytic activity of the ZIKV E+102CpG variant. In accordance with in vitro results, in ovo, oncolytic activity also depended on the viral CpG content: while GSC 528-derived tumors infected with ZIKV E/NS1+176CpG showed only moderate volume reduction (3.4 times), the ZIKV E+102CpG variant showed oncolytic activity with high tumor volume reduction comparable to WT ZIKV (13–16 times; Figure 5e). This dissonance of different CpG-recoded variants demonstrates that oncolytic activity of a virus can be tuned by adjusting the number of de novo introduced CpG dinucleotides within a viral genome.

Oncolytic activity of WT and recoded ZIKV variants varied in GSCs derived from different patients and with different cell phenotypes. While ZIKV variants showed oncolytic activity in GSC 528 in vitro and in ovo, GSC 157 was resistant to oncolytic activity. A recent study showed that ZIKV preferentially infects and kills GSCs in a SOX2-dependent manner [56]; however, our data suggest that SOX2-negative GSCs (similar to GSC 528) can be susceptible to ZIKV oncolytic activity and in vitro SOX2-positive GSCs (similar to GSC 157) can be resistant. The heterogeneity in susceptibility and resistance of GSCs from different patients to chemotherapy is well-known [26]. The resistance of some GSCs to ZIKV oncolytic activity is an important finding emphasizing the need for personalized oncolytic therapy or a strategy to overcome resistance mechanisms in GSCs.

Further safety and efficacy studies are needed to highlight limitations and advantages of the CpG recoding approach for oncolytic viruses. The important consideration for therapy is the safety of oncolytic viruses for patients and public health. Recoded oncolytic candidates with hundreds of extra CpG dinucleotides most probably will show rare or no reversion to virulence; in support, the stability of de novo introduced CpGs in the ZIKV genome during in vitro and in vivo infection has been demonstrated in our recent study [7]. Moreover, in contrast to the WT virus, the present most promising ZIKV E+102CpG oncolytic candidate previously showed an excellent safety pattern in a mouse pregnancy model [7]. Another potential perspective for the safe CpG recoding oncolytic approach is in combination with other established oncolytic strategies; for example, CpG recoding can serve as an additional safety level. A small number of nucleotide mutations or deletions may determine attenuation in efficient oncolytic viruses—e.g., in a modified ZIKV with only 10 nucleotide deletions in 3′ UTR [57]. This reliance on a small number of critical mutations/deletions in oncolytic viruses might lead to reversion to virulence during highly efficient replication in GSCs that are more conducive to infection than nonmalignant host cells. One more example—Seneca Valley virus—is a promising oncolytic candidate that does not cause infection in humans but poses a significant threat to livestock [58]. A rational strategy for CpG recoding may reduce the potential for zoonotic spillover of oncolytic viruses and outbreaks in livestock.

Towards efficacy, novel therapeutic approaches against glioblastoma are formulated to modulate the immune response towards the tumor and the surrounding microenvironment [59]. In DNA molecules, CpG dinucleotides can directly activate B cells, natural killer cells, dendritic cells, monocytes, and macrophages through TLR9 stimulation [60]; introduced CpG dinucleotides in synthetic RNA molecules may also activate cellular immune responses; however, the mechanisms of activation remain unclear [61]. CpG islands have been inserted into a double-stranded DNA adenovirus genome to increase adjuvancy that resulted in enhanced TLR9-stimulation for increased antitumor activity [62]. Whether the increased CpG content in recoded RNA viruses leads to local brain immune activation and whether that augments oncolytic efficacy against glioblastoma remain to be studied.

The limitation of this pilot study is that as in previous ZIKV oncolytic studies [8,9,14], we had GSCs from only a limited number of patients. However, we established an in vitro and in ovo experimental toolbox with ZIKV-sensitive (GSC 528) and ZIKV-resistant (GSC 157) cells. This experimental system can be used to identify GSC factors that determine resistance to ZIKV oncolytic activity.

Collectively, our results show the potential of the CpG recoding approach for oncolytic therapy. These findings should encourage further research towards a better understanding of interactions between CpG-recoded viruses, the tumor, tumor environment, and host responses. This understanding may deliver adaptable CpG-recoding technology for safe and efficient oncolytic viruses.

## Supplementary Materials

The following are available online at https://www.mdpi.com/1999-4915/12/5/579/s1, Figure S1: Infection kinetics in nonmalignant human brain cells (HMC3 and NPC) and tumor glioblastoma stem cells (528 and 157) after inoculation at an multiplicity of infection of 0.01.; Table S1: Cytosine–phosphate–guanine (CpG) dinucleotide composition in Zika virus (ZIKV) variants.
