# Supplementary material for: Zika Virus with Increased CpG Dinucleotide Frequencies Shows Oncolytic Activity in Glioblastoma Stem Cells
Source: Viruses. 2020 May 25;12(5):579. doi: 10.3390/v12050579 (PMC7290362; doi:10.3390/v12050579)
Supplement: Supplementary file 1 [file viruses-12-00579-s001.pdf]

viruses

Supplementary Materials: Zika Virus with Increased CpG Dinucleotide Frequencies Shows Oncolytic Activity in Glioblastoma Stem Cells

Ivan Trus, Nathalie Berube, Peng Jiang, Janusz Rak, Volker Gerdts and Uladzimir Karniychuk

Table S1. CpG dinucleotide composition in ZIKV variants.

Genomic region	ZIKV variant	Number of CpGs
E	Wild-type	35
	Permuted	35
	E+32CpG	67
	E+102CpG	137
	E/NS1+176CpG	137
NS1	Wild-type	25
	Permuted	25
	E+32CpG	25
	E+102CpG	25
	E/NS1+176CpG	99
ORF [1]	Wild-type	316
	Permuted	316
	E+32CpG	348
	E+102CpG	418
	E/NS1+176CpG	492

[1] ORF: open reading frame

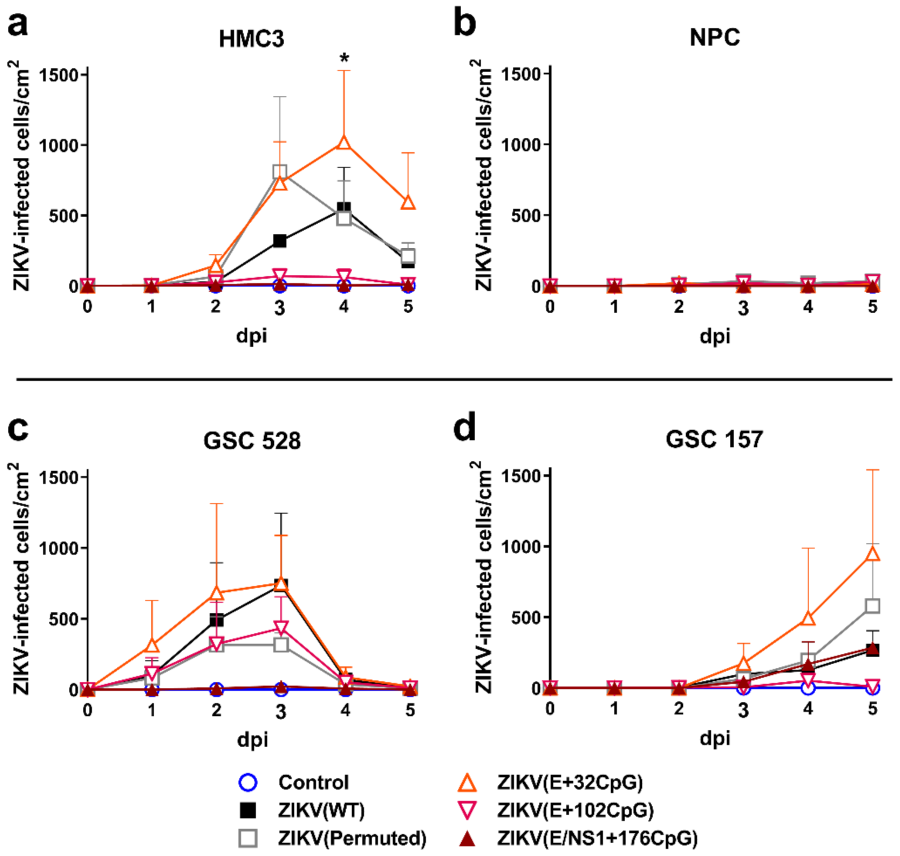

Figure S1. Infection kinetics in nonmalignant human brain cells (HMC3 (**a**) and NPC (**b**)) and tumor glioblastoma stem cells (528 (**c**) and 157 (**d**)) after inoculation at an MOI of 0.01. The 96-well plates with cell monolayers were stained with ZIKV-specific Abs and infected cells were counted in the whole well with bright-field microscopy at 200×. Whiskers represent standard error of the mean (SE) from three biologically independent replicates with three technical replicates. "dpi"–days post-inoculation. The asterisk (*) indicates p < 0.05 vs. WT: (**a**) E+102CpG and E/NS1+176CpG at 4 dpi.
